# Clinical Aspects and Management of Levodopa-Induced Dyskinesia

**DOI:** 10.1155/2012/745947

**Published:** 2012-06-03

**Authors:** Nicola Tambasco, Simone Simoni, Erica Marsili, Elisa Sacchini, Donatella Murasecco, Gabriela Cardaioli, Aroldo Rossi, Paolo Calabresi

**Affiliations:** ^1^Clinica Neurologica, Azienda Ospedaliera—Università di Perugia, S. Andrea delle Fratte, 06156 Perugia, Italy; ^2^IRCCS Fondazione S. Lucia, Via Ardeatina 306, 00179 Roma, Italy

## Abstract

In Parkinson's disease, one of the most troublesome dilemmas is the treatment of levodopa-induced dyskinesia. After a few years, chronic treatment with levodopa is associated with the development of dyskinesias. Strategies to delay or to reduce dyskinesias are based on the change of levodopa dosing or the early use of dopamine agonists. Dopamine agonists with different pharmacological profile are available. Our paper was aimed to analyse the clinical impact and the management of dyskinesias with dopamine agonists.

## 1. Introduction

Four decades after its introduction, levodopa remains the most effective agent to improve motor symptoms in PD, but chronic use is associated with the emergence of motor fluctuations, defined as a loss of clinical benefit before the next levodopa dose (wearing off), abnormal involuntary movements (dystonia, chorea, and athetosis—collectively referred to as dyskinesia) [1, 2], and nonmotor complications, as behavioural and cognitive changes [3]. Levodopa is initially well tolerated in most of the cases and allows a substantial improvement of motor performances despite its erratic pharmacokinetics [1, 4]. With the disease progression, therapeutic window of levodopa narrows, and the duration of each dose shortens. Motor fluctuations usually precede dyskinesias [5], and it has been observed that the development of one is a risk factor for the development of the other [5].

Although more commonly associated with levodopa, dyskinesias can also occur with dopamine agonist monotherapy [6–8]. The development of dyskinesia in some patients treated with dopamine agonists that have relatively long half-lives (ropinirole, 6 h; pramipexole, 8 h) or very long half-lives (cabergoline, 68 h) suggests that, to some extent, even dopamine stimulation provided in a continuous fashion can cause dyskinesias.

## 2. Epidemiology and Clinical Aspects of Motor Complications

The three most important risk factors positively associated with increased occurrence of dyskinesias are younger age at disease onset [9, 10], longer disease duration [11, 12], and longer duration of pulsatile dopaminergic treatment (typically, levodopa) [13, 14]. The first two factors are interrelated and almost all patients with early-onset PD [15] develop dyskinesias, whereas they are less frequent in patients with late-onset PD [16]. PD patients with early disease onset have a high probability to carry mutations for monogenic PD forms, and therefore, early onset and genetic predisposition are two overlapping and possibly interrelated risk factors. Other risk factors associated with increased risk of dyskinesias are female gender [17, 18] and the occurrence of specific polymorphisms for dopamine receptors or dopamine transporters [19–21].

Dyskinesias more commonly appear as choreiform, but in some cases, they may resemble dystonia, myoclonus, or other movement disorders. Peak-dose dyskinesias are the most common type of dyskinesia. They occur during peaks of levodopa-derived dopamine in the brain, when the patient is otherwise experiencing a beneficial response (the “on” state). Peak-dose dyskinesias worsen with increases in dopaminergic dose and lessen with its reductions. In certain cases, dyskinesias seem to appear with a more particular pattern, as dyskinesia-improvement-dyskinesia. This is termed diphasic dyskinesia, and it tends to occur when levodopa-derived dopamine concentrations are increasing or decreasing, whereas the clinical condition of the patient turns “on” and “off” [22]. Diphasic dyskinesias are typically displayed with large-amplitude stereotypic, rhythmic, and repetitive movements, more often of the legs, that may be associated with Parkinsonian features in other body regions. In extreme cases, patients treated with levodopa can cycle between “on” periods, which are complicated by disabling dyskinesias, and “off” periods in which Parkinsonism is uncontrolled and the patient is akinetic and frozen.

Motor complications occur in about 50% of patients with PD who have been in therapy with levodopa for more than 5 years, and in almost 100% of patients with young-onset disease [23, 24]. Achieving an acceptable clinical control once these motor fluctuations have appeared is usually a relatively simple matter, nearing together the levodopa doses or adding medications that reduce “off” time. However, when a patient develops peak-dose dyskinesias too, it becomes difficult to smooth the clinical response. Although for many patients, dyskinesias are not disabling, they create a barrier to adequate treatment of fluctuations and Parkinsonian symptoms.

## 3. Pathophysiology of Dyskinesias

A primary condition in LID pathophysiology is the presence of dopaminergic cell loss in substantia nigra [25–27]. The nonappearance of dyskinesia in normal humans chronically treated with levodopa (i.e., mistaken diagnosis [28]) and its rapid emergence in PD patients either with late diagnosis or a young onset, where denervation is high at diagnosis [15, 29, 30], heavily support this theory. Moreover, the progression of nigral denervation seems to be closely related with the lowering of the dyskinesia onset threshold in MPTP-exposed primates [31]. Nonetheless, denervation cannot be the unique factor responsible for dyskinesia, whereas not all patients with advanced illness and extensive nigral denervation develop dyskinesia when treated with levodopa [32, 33]. Thus, a chronic dopaminergic stimulation on a denervated substantia nigra induces a process of sensitisation such that each following administration modifies the response to subsequent dopaminergic treatments. This process, called priming, increases over time of treatment the chance of eliciting dyskinesias and, once dyskinesias have been established, their severity. The priming process, which is responsible for the insidious evolution of dyskinesias over time of treatment, is associated with changes in receptors for dopamine or other neurotransmitters [34, 35]. A crucial role has been postulated for both dopamine receptors and NMDA glutamate receptors in the induction of priming; this mechanism could be regarded as an increased responsiveness of postsynaptic striatal dopamine receptors (mainly D_1_-like), which are activated in conjunction with glutamatergic inputs [1]. Dyskinesias are probably generated by a persistent enhancement of the responsiveness of striatal medium-sized spiny neurones to dopaminergic treatment. This is an aftermath of dopamine depletion and is associated with overexpression of specific components of the signal transduction machinery. If protracted, this condition may ultimately lead to long-term changes in gene expression, which will permanently affect the function of striatal medium spiny neurones [36]. Following priming, the development of dyskinesias largely depends on two additional factors, the pulsatile administration of levodopa (or another short-acting dopaminergic agent) and the severity of dopaminergic denervation in the striatum. The latter plays an important role in setting the threshold required in developing dyskinesias [37]. A direct relationship between the severity of striatal denervation and the time required to develop dyskinesias has been demonstrated in PD patients [38] and has been indirectly confirmed by the finding that patients with dopa-responsive dystonia, who have Parkinsonism without nigrostriatal denervation, uncommonly develop dyskinesias [39].

In early PD patients, levodopa-derived dopamine is packaged into synaptic vesicles by vesicular monoamine transporter 2 (VMAT-2), stored, and released in both tonic and phasic bursts in response to impulse flow [40, 41], in order to preserve dopamine receptors from levodopa plasma concentration fluctuations and, therefore, to maintain physiological dopaminergic transmission [42, 43]. With the progression of the disease, and the striatal dopaminergic cell loss, the formation of dopamine from levodopa and its storage capacity are increasingly compromised, and the response to levodopa becomes dominated by its pharmacokinetic characteristics and general bioavailability [4]. Thus, in advanced PD, peak concentrations of drug in plasma become coincidental with the expression of dyskinesia. As observed in animal models, the continuous release of dopamine leads to improvements in motor function and, together, to a marked reduction in the expression of involuntary movements [44]. These studies support the clinical findings that the continuous intravenous or intraduodenal administration of levodopa or the continuous subcutaneous or intravenous infusion of apomorphine results in improved motor response but also with a marked reduction of dyskinesia [45, 46].

Other mechanisms are involved to explain the underlying cause and expression of dyskinesia. Although dopamine agonists when used as monotherapy in early PD are associated to a lower incidence of dyskinesia, involuntary movements are still observed, reflecting some kind of activity at the postsynaptic dopamine receptor level, as dopamine agonists are not dependent on the presence of presynaptic terminals.

Subtle changes in D_1_ and D_2_ receptor density as well as the complex interaction between receptor activation and synaptic plasticity [1] have been proposed as playing significant roles in dyskinesia induction and expression. Although the exact molecular mechanisms of LID still remain to be fully elucidated, exaggerated signalling of the striatal D_1_ [47–49], the reduction of the modulating function of D_2_/D_3_ receptors [42, 43, 50–52], and the interaction between D_2_ and A2A adenosine receptors [53] have been implicated in both rodents and primates, suggesting that a normalisation of signalling may be beneficial in the treatment of dyskinesia.

In clinical practice, postsynaptic mechanisms can be partially explained by the dopamine agonists capability to prime for involuntary movements. Switching from a chronic dopamine agonist administration that usually results in a low expression and intensity of dyskinesia to an equivalent dose of levodopa in fact immediately results in the appearance of dramatic involuntary movements [54, 55]. These findings suggest that dopamine agonists principally prime for, but less commonly express dyskinesia. Vice versa, when considering the expression of dyskinesia in patients with PD with a history of levodopa exposure, switching to a dopamine agonist after the introduction of levodopa, established dyskinesia still occurred [56]. Moreover, patients receiving a combination of levodopa and the dopamine D_2_/D_3_ agonist pramipexole showed a level of dyskinesia that was greater than the additive effect of the individual drug [57]. Once established, dopamine agonists produce the same pattern of dyskinesia although its intensity is reduced, suggesting that agonists do not express dyskinesia to the same extent as levodopa [54, 55, 58]. Both the lower priming for dyskinesia and the lower expression of involuntary movements by dopamine agonists may be a reflection of their more specific pharmacology compared to levodopa.

## 4. Reducing or Delaying Parkinsonian Dyskinesias

Any type of exogenous dopaminergic stimulation in a denervated striatum can cause dyskinesias [59], but pulsatile stimulation produced by short-acting drugs (as typically occurs with levodopa) particularly favours their occurrence [60]. The expression LID is still currently used, although levodopa is not the only drug causing dyskinesias in PD patients [61]. Based on published series, it has been estimated that PD patients treated for less than 5 years have a 11% risk of developing dyskinesias, those treated for 6–9 years have a risk of 32%, whereas patients treated for more than 10 years have a risk of 89% [13].

Levodopa, however, seems to be the most important factor in inducing dyskinesia expression in chronically treated PD patients; therefore, it appears that the benefit of initial treatment with a dopamine agonist in lowering the incidence of dyskinesias is related to the ability of the agonist to delay the need for levodopa [12, 62]. Moreover, experimental data suggest that the administration of long-acting dopamine agonists results in significantly less dyskinesia than does levodopa [63, 64] and other short-acting agents administrated in a pulsatile fashion [65]. However, once a long-acting agonist is administered to animals already primed to exhibit dyskinesias with levodopa, the resultant dyskinesias are comparable to those seen in the levodopa group [63]. Clinical studies randomly assigning patients to initial treatment with a dopamine agonist or levodopa have shown a lower risk for dyskinesias in the groups treated with pramipexole [7], ropinirole [8, 12], bromocriptine [66, 67], pergolide [68], and cabergoline [6]; nevertheless, once levodopa was added, the rate of development of dyskinesias was similar in both groups.

One therapeutic strategy that has been tried in this sense is to use higher doses of a dopamine agonist to reduce both the total daily levodopa dose and its frequency [69] or to gradually substitute a dopamine agonist for levodopa [70]. Unfortunately, these strategies are unsatisfactory and typically reduce dyskinesias at the expense of less control of Parkinsonian symptoms. Indeed, the evidence that early levodopa exposure adversely affects the course of disease and leads to disabling dyskinesias and motor fluctuations constituted the rationale for the initial treatment with dopamine agonist.

## 5. Different Profile and Efficacy of Dopamine Agonists in Reducing Dyskinesia

**Figure 1:**
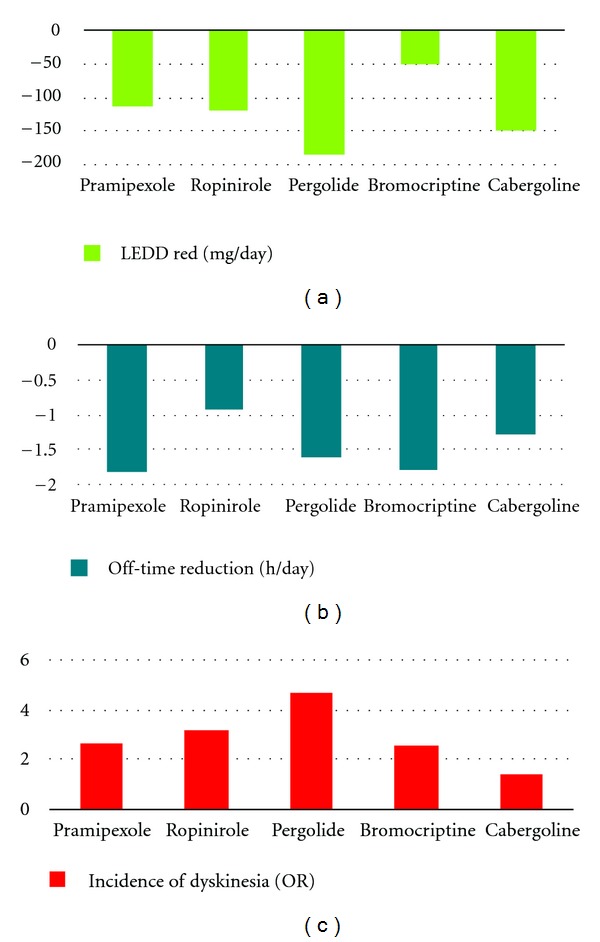
Effects of dopamine agonists on (a) reducing off time, (b) reducing levodopa daily dose, and (c) inducing dyskinesia.

In order to create a valid alternative to levodopa, and with the aim of eliminating its related complications, many different drugs acting on dopaminergic receptors have been developed and studied during the last years. They have different metabolism, plasma half-life, affinity to receptors subtypes, excretion, and routes of administration (Table 1). Moreover, these drugs have different efficacies on reducing the incidence of dyskinesia, improving motor symptoms, and reducing the daily levodopa dose (Table 2, Figure 1).

Initially dopamine agonists have been used as adjuvant therapy to improve levodopa-induced complications, but once their effects on delaying the need for levodopa have been demonstrated, they have often been prescribed before the introduction of levodopa. Patients receiving dopamine agonists rather than levodopa as initial monotherapy showed a reduced risk for developing dyskinesias [7, 8, 12, 62, 72–76] (Table 3).

### 5.1. Dopamine Agonists Monotherapy and the Risk of Dyskinesia

The CALM-PD trial (Comparison of the Agonist Pramipexole versus Levodopa on Motor Complications of Parkinson's Disease) was a randomised controlled trial evaluating the risk of developing dyskinesias in patients with early PD initially treated with either pramipexole or levodopa. After 24 months, pramipexole-treated patients were receiving pramipexole plus levodopa, compared with levodopa alone. A minority of pramipexole-treated patients reached the endpoint of time to first occurrence of wearing off, dyskinesias, or on-off motor fluctuations (27.8% versus 50.7%, *P* < 0.001). Moreover, a significantly lower incidence of dyskinesias (9.9% versus 30.7%, *P* < 0.001) also has been demonstrated in patients in the pramipexole group. However, after a mean 6-year followup, >90% of patients were receiving levodopa therapy regardless of their initial treatment assignment. Compared to those taking pramipexole, patients initially treated with levodopa had significantly more dyskinesias (20.4% versus 36.8%), but there was no difference between groups in the incidence of disabling or painful dyskinesias [62, 74]. Interestingly, 5 subjects taking pramipexole developed dyskinesias before the supplemental levodopa, and 4 of them had no prior levodopa exposure [73]. No significant difference in the Lang-Fahn activities of daily living dyskinesia score was observed (1.3 versus 1.1 with pramipexole, *P* < 0.06) [7, 62, 72–74].

In a randomised, double-blind 5-year study of patients with early PD, the risk of developing dyskinesias after initial monotherapy with ropinirole was less than with levodopabenserazide (hazard ratio (HR), 2.82 (1.78, 4.44); *P* < 0.001) [8]. However, many of these patients eventually required supplemental levodopa to control the symptoms of the disease [8, 12]. When patients receiving ropinirole monotherapy required the addition of levodopa, the risk for developing dyskinesias increased and then did not differ significantly from that associated with levodopa alone [12]. The use of ropinirole as monotherapy, with only later addition of levodopa, delayed the onset of dyskinesias by up to 3 years, although it was associated with a higher incidence of neuropsychiatric complications than levodopa monotherapy.

Apomorphine, a subcutaneous nonergolinic dopaminergic agent, has been studied in 2 retrospective chronic monotherapy trials in which no oral anti-parkinsonian therapies were permitted from the time the pump was turned on in the morning until it was turned off in the evening [77, 78]. The mean maximum reduction of dyskinesia per patient was 64% (*P* < 0.005), and the mean time to achieve maximal dyskinesia improvement was 12.1 months.

Lisuride, another subcutaneously administered dopamine agonist, given as a continuous daytime infusion via pump, has been utilised as a strategy for minimising dyskinesias in 40 patients with advanced, levodopa-responsive PD characterised by motor fluctuations and dyskinesias [109]. After 4 years, the lisuride-treated patients had improved their baseline dyskinesia scores (measured by AIMS) by 49% (*P* < 0.0001), whereas the levodopa-treated patients had worsened their scores by 59% (*P* < 0.0001). 

### 5.2. Long-Acting Dopamine Agonists and the Risk of Dyskinesia

In animal-model studies, the long-acting dopamine agonists have been demonstrated to prevent or reduce the onset time for LIDs. In a study of monkeys with MPTP-induced parkinsonism, small doses of subcutaneously administered cabergoline, a D_2_-selective dopamine agonist with a relatively long half-life, were added as adjuvant therapy to orally administered levodopa/benserazide (100/25 mg) for 1 month, showing significantly lower dyskinesia scores (sum for all body segments) than when levodopa/benserazide was given alone for 1 month (*P* < 0.01).

A report on the effect of cabergoline compared to levodopa showed a reduced incidence of dyskinesias [110]. Nevertheless, more recently, an increased incidence of dyskinesia and confusion in patients treated with bromocriptine was reported [111].

### 5.3. Differences among Drugs in Adjuvant Therapy

A recent systematic meta-analysis, which performs indirect comparisons among three classes of drugs, including nondopaminergic agents as catechol-O-methyl transferase inhibitors (COMTIs) or monoamine oxidase type B inhibitors (MAOBIs), used as add-on (adjuvant) treatment to levodopa therapy in PD patients with motor complications, suggests that dopamine agonists may provide more effective symptomatic control [112]. 

#### 5.3.1. Off-Time Reduction

There is no (or little) evidence of a difference across the different dopamine agonists for the overall reduction in off-time [pramipexole (−1.81 hours/day, CI −2.19 to −1.43; *P* < 0.00001); bromocriptine (−1.78 hours/day, CI −2.91 to −0.65; *P* = 0.002); pergolide (−1.60 hours/day, CI −2.57 to −0.63; *P* = 0.001); cabergoline (−1.29 hours/day, CI −1.89 to −0.69; *P* < 0.0001); ropinirole (−0.93 hours/day, CI −1.53 to −0.33; *P* = 0.002)] [112].

#### 5.3.2. Levodopa Daily Dose Reduction

The largest reduction was with pergolide (−183.90 mg/day, CI −259.09 to −72.71; *P* = 0.001), though this was based on data from just one trial [71]. Cabergoline reduced the required levodopa dose by 149.60 mg/day (CI −208.79 to −90.41; *P* < 0.00001), ropinirole by 119.81 mg/day (CI −150.63 to −89.00; *P* < 0.00001), pramipexole by 114.82 mg/day (CI −143.01 to −86.64; *P* < 0.00001), and bromocriptine by 52.17 mg/day (CI −95.16 to −9.18; *P* = 0.02) [112].

#### 5.3.3. UPDRS Scores Improvement

The agonist pramipexole appeared to produce larger improvements for UPDRS motor score (−6.31 points, CI −7.69 to −4.93; *P* < 0.00001) compared to ropinirole (UPDRS motor: −4.80 points, CI −7.32 to −2.28; *P* = 0.0002) and cabergoline (UPDRS motor: −1.74 points, CI −3.78 to 0.30; *P* = 0.09) [112].

#### 5.3.4. Incidence of Dyskinesia

The analysis included 6476 participants, which represented 85% of the 7590 randomised participants included in the meta-analysis. Compared to placebo, the incidence of dyskinesia was increased with adjuvant therapy. The incidence of dyskinesia was greatest with pergolide (OR 4.64, CI 3.09 to 6.97; *P* < 0.00001), although the data were obtained from just one trial [71], followed by ropinirole (OR 3.21, CI 1.98 to 5.21; *P* < 0.00001), pramipexole (OR 2.63, CI 2.01 to 3.42; *P* < 0.00001), bromocriptine (OR 2.52, CI 1.42 to 4.48; *P* = 0.002), and cabergoline (OR 1.44, CI 0.96 to 2.16; *P* = 0.08) [112].

Though this meta-analysis indirectly compares several series on dopaminergic agents as adjuvant treatment, the need of large randomised studies that directly compare different agents administered as monotherapy with patient-rated overall quality of life and health economic measures as primary outcomes is recommended.

## 6. Alternative Treatments to Reduce Dyskinesia

As seen earlier, the primary therapeutic strategy for managing LIDs in PD patients is to delay their occurrence through delaying the introduction of levodopa therapy administering dopaminergic agents.

Once dyskinesias have occurred, other strategies should be attempted: (1) substitution of immediate release for controlled-release levodopa. The immediate-release preparation is easier to adjust, as onset of its effects is sooner, and duration of action (and dyskinesias) is shorter than with controlled-release preparations. For the same reason, agents that prolong the half-life of levodopa, such as entacapone, should be stopped; (2) discontinuation of other therapy that may embitter dyskinesias, as dopamine agonists or other factors delaying dopamine degradation as selegiline and rasagiline; (3) incrementation of the number of administrations of levodopa, in lower doses; (4) addition of an antidyskinetic agent as amantadine, an NMDA receptor antagonist. Diphasic dyskinesias that may manifest at the beginning and the end of a dosing cycle should be managed by utilising more frequent doses of levodopa, and the therapy should be sewed on the patient [113].

### 6.1. Amantadine

The NMDA receptor-binding and neurotoxic effects of excessive glutamate have led to the hypothesis that an NMDA antagonist may have antidyskinetic effects and reduce the severity of LIDs. Amantadine has been studied as adjuvant treatment in levodopa-treated patients experiencing motor complications, including dyskinesias, with the aim of reducing these effects without worsening Parkinsonian symptoms [114–117]. Three randomized placebo-controlled crossover clinical studies in a group of 53 PD patients showed a reduction (up to 60%) in the severity of LIDs after challenge with acute levodopa administration, without impacting the beneficial effects of levodopa on motor function.

### 6.2. Clozapine

It is an atypical antipsychotic that has been assessed for the treatment of drug-induced psychosis in PD. It may also be effective in decreasing dyskinesias [70], and a few studies have focused on its antidyskinetic effect [118, 119].

### 6.3. Intraduodenal Levodopa

It provides direct delivery of levodopa to the duodenum and jejunum. The method involves insertion of a permanent access tube in the abdominal wall by percutaneous endoscopic gastrostomy. Several clinical studies have been conducted using this approach, demonstrating significant reductions in “off” time and dyskinesia after 6 months. It may be an option for patients with marked fluctuations and dyskinesia in whom deep-brain stimulation (DBS) is contraindicated or not possible due to advanced age, or it may provide an alternative to DBS.

### 6.4. Surgical Treatment

Patients with PD who may benefit from surgery include those who have substantial dyskinesias unresponsive to medication adjustments, are levodopa responsive, do not have dementia, and do not have neuropsychiatric impairment [80]. DBS is the most frequently performed surgery for PD in North America [80]. In patients with advanced PD, DBS of the globus pallidus interna (GPi) or the subthalamic nucleus (STN) has been shown to reduce dyskinesia severity by up to 89% [120, 121] and to reduce the duration of dyskinesias by 86% [122]. It provides significant improvement in Parkinsonian motor features and allows a reduction of dyskinesias, in part through the subsequent reduction of levodopa [123, 124].

## Figures and Tables

**Table 1:** Pharmacological characteristics of dopamine agonists.

	Pramipexole	Ropinirole	Rotigotine	Pergolide	Bromocriptine	Cabergoline	Apomorphine	Lisuride
D_1_	0	0	+	+	−	0/+	+++	−
D_2_	+++	+++	+++	++++	++	+++	++	++++
D_3_	++++	++++	++++	+++	++	++	++++	+++
Type	Nonergot	Nonergot	Nonergot	Ergoline	Ergoline	Ergoline	Morphine deriv.	Ergoline
Routes	os	os	td	os	os	os	sc	sc
Metabolism	—	Hepatic	Hepatic	Hepatic	?	Hepatic	Hepatic	Hepatic
Elimin.	Urine	Urine	Urine/fecal	Urine/fecal	Fecal	Fecal/urine	Urine/fecal	Urine/fecal
Half-life (h)	8–12	5–6	5–7	27	12–14	63–69	40 min.	2

td: transdermal; sc: subcutaneous.

**Table 2:** Adjuvant therapy versus placebo.

	Pramipexole	Ropinirole	Pergolide^∗^	Bromocriptine	Cabergoline
Off-time reduction (h/day)	−1.81	−0.93	−1.60	−1.78	−1.29
LEDD red (mg/day)	−114.82	−119.81	−183.90	−52.17	−149.60
UPDRS ADL reduction (pts)					−1.78
UPDRS III reduction (pts)		−4.80			−1.74
Incidence of dyskinesia (OR)	2.63	3.21	4.64	2.52	1.44

^∗^Based on data from just one trial [71].

**Table 3:** Series on adjuvant therapy with dopamine agonists^∗^. In italic, dyskinesia evaluation.

Author	Duration	Characteristics of participants	Interventions	Primary outcomes	Secondary outcomes
*Poewe et al. *[79]	* (6 months)*	*N: 302; MFs. Mean duration of PD: 8.5 y*	*Pramipexole (n* = 201*) versus placebo (n* = 101)	*Disability; motor complications; on/off time*	*SE*
*Pahwa et al. *[80, 81]*; Sethi et al.* [82, 83];* Stacy et al. *[84]; *Stocchi et al.* [85, 86]	* (24 weeks)*	*N: 393; MFs. Mean duration of PD: 8.6 y*	*Ropinirole (24-h) (n* = 202*) versus placebo (n* = 191)	*Disability; patient-rated QoL; on/off time; levodopa dose*	*SE* *Depression sleep scales*
*Oertel et al.* [87]; *Pogarell et al.* [88]	* (32 weeks)*	*N: 363 (354 analysed); MFs. Mean duration of PD: 7.8 y*	*Pramipexole (n* = 180*) versus placebo (n* = 183)	*Disability; off time; levodopa dose*	*SE*
Wong et al. [89]	(15 weeks)	*N*: 150; Mean duration of PD: 4.4 y	Pramipexole (*n* = 73) versus placebo (*n* = 77)	Disability; off time	SE
*Musch and Bonura* [90]	* (24 weeks)*	* N: 218; on levodopa. Mean duration of PD: NA*	*Cabergoline (n* = 145*) versus placebo (n* = 73)	*Disability; off time; levodopa dose*	*SE*
*Pinter et al. *[91]	* (11 weeks)*	*N: 78; MFs. Mean duration of PD: 8.2 y*	*Pramipexole (n* = 34*) versus placebo (n* = 44)	*Disability; off time; levodopa dose*	*SE*
*Wermuth *[92]	* (12 weeks)*	* N: 69; MFs. Mean duration of PD: 10 y (range: 3–27 y)*	*Pramipexole (n* = 36*) versus placebo (n* = 33)	*Disability; motor complications; off time; levodopa dose*	*SE*
*Lieberman et al. *[93];* Weiner et al. *[94]	* (32 weeks)*	* N: 360; MFs. Mean duration of PD: 9.2 y*	*Pramipexole (n* = 181*) versus placebo (n* = 179)	*Disability; on/off time; levodopa dose*	*SE*
*Guttman *[95]	* (9 months)*	*N: 247; MFs. Mean duration of PD: 7 y (range: 0.67–36 y)*	*Pramipexole (n* = 79*) versus bromocriptine (n* = 84*) versus placebo (n* = 83)	*Disability; off time*	*SE*
*Kreider et al.* [96];* Lieberman et al. *[97]	* (6 months)*	*N: 149; predictable MFs. Mean duration of PD: 9 y*	*Ropinirole (n* = 95*) versus placebo (n* = 54)	*Disability; motor complications; off time; levodopa dose*	*SE*
*Rascol et al. *[98, 99]	* (12 weeks)*	* N: 46; not optimally controlled with levodopa. Mean duration of PD: 8 y*	*Ropinirole (n* = 23*) versus placebo (n* = 23)	*Disability; motor complications; off time*	*SE*
Steiger et al. [100]	(6 months)	*N*: 37; MFs. Mean duration of PD: 12.8 y (range: 3–33 y)	Cabergoline (*n* = 19) versus placebo (*n* = 18)	Disability; motor complications; off time; levodopa dose	SE
*Hutton et al. *[101]; *Lieberman and Hutton *[102];* Schoenfelder et al. *[103]	* (24 weeks)*	* N: 188; MFs. Mean duration of PD: 10.6 y (range: 2–30 y)*	*Cabergoline (n* = 123*) versus placebo (n* = 65)	*Disability; on/off time; levodopa dose*	*SE*
*Olanow et al. *[104]	* (6 months)*	* N: 376; MFs. Mean duration of PD: 10.9 y*	*Pergolide (n* = 189*) versus placebo (n* = 187)	*Disability; motor complications; off time; levodopa dose*	*SE*
Temlett et al. [105]	(5 weeks)	*N*: 44 (40 analysed); Mean duration of PD: 13.4 y	Bromocriptine (*n* = 22) versus placebo (*n* = 18)	Levodopa dose	SE
*Toyokura et al. *[106]	* (8 weeks)*	*N: 222; not optimally controlled with levodopa. Mean duration of PD: 6.6 y*	*Bromocriptine (n* = 114*) versus placebo (n* = 108)	*Motor complications; on/off time*	*SE*
Schneider and Fischer [107]	(4 weeks)	*N*: 40; not optimally controlled with levodopa. Mean duration of PD: 9.1 y	Bromocriptine (*n* = 20) versus placebo (*n* = 20)	On/off time; levodopa dose	
Jansen [108]	(5 months)	*N*: 23; not optimally controlled with levodopa. Mean duration of PD: 8.7 y	Bromocriptine (*n* = 12) versus placebo (*n* = 11)	Disability	

^∗^Performed on PD patients, parallel groups, double blind.

MFs: motor fluctuations; SE: side effects.
